# Case report: Weil’s disease with multiple organ failure in a child living in dengue endemic area

**DOI:** 10.1186/s13104-016-2210-4

**Published:** 2016-08-15

**Authors:** Dewi Lokida, Arif Budiman, Udjiani Edi Pawitro, M. Hussein Gasem, Muhammad Karyana, Herman Kosasih, Sophia Siddiqui

**Affiliations:** 1 Tangerang District Hospital, Tangerang, Indonesia; 2 Kariadi Hospital, Semarang, Indonesia; 3 Indonesia Research Partnership on Infectious Diseases (INA-RESPOND), Jakarta, Indonesia; 4 National Institute of Health Research and Development (NIHRD), Jakarta, Indonesia; 5 US, National Institute of Allergy and Infectious Disease, Bethesda, USA

**Keywords:** Weil’s disease, Leptospirosis, Children, Dengue

## Abstract

**Background:**

There were few reports in the literature of Weil’s disease with multiple organ failures, especially in children living in dengue endemic areas.

**Case presentation:**

A 12-year-old child was admitted to Tangerang district hospital with a provisional diagnosis of dengue infection. On the third day of hospitalization, dengue diagnostic tests were negative. As fever still remained and was followed by jaundice, decreasing hemoglobin, increasing bilirubin with abnormal value of liver enzymes; other causes of disease were investigated. Leptospirosis was confirmed by rapid IgM test (SD^®^) for leptospira; and micro-agglutination test which indicated *Leptospira serogroup bataviae* infection. The patient developed Weil’s disease during the course of illness. Renal function was back to normal on the 21st day of hospitalization, while hemoglobin and bilirubin returned to normal three weeks after discharged.

**Conclusions:**

Our report highlights the importance of considering leptospirosis as a differential diagnosis in children with acute febrile illness; even when the signs and symptoms for the more common diagnoses such as dengue or typhoid fever were pathognomonic. A normal leukocyte count with neutrophilia and negative dengue NS1, dengue IgM, and *Salmonella typhi* IgM on admission should raise suspicion of leptospirosis, and prompt diagnostic assays for leptospirosis should be conducted.

**Electronic supplementary material:**

The online version of this article (doi:10.1186/s13104-016-2210-4) contains supplementary material, which is available to authorized users.

## Background

Leptospirosis is a zoonosis caused by *Leptospira interrogans* complex [1]. Humans are infected through direct exposure to an infected animal, or indirectly via contaminated soil or water. *Rattus norvegicus* (brown rat) is the most important source of infection [1]. The clinical presentations range from subclinical to fatal (e.g.: Weil’s disease) [1]. Severe cases are more frequent in adults than children [2, 3].

In Indonesia, leptospirosis was reported annually in 3–7 of its 34 provinces. The total number of cases increased from 335 in 2009 to 651 in 2013 [4]. As Indonesia is a populous country, these numbers may be under-estimated for several reasons. Firstly, leptospirosis is clinically undistinguishable from other infectious diseases, such as rickettsiosis, dengue, or typhoid fever, especially during the early stages of illness. Secondly, diagnostic tests are not easily available. Lastly, leptospirosis is often not considered, especially in children.

## Case presentation

A 12-year-old boy presented to Tangerang district hospital, on 12 February 2015. Tangerang is a suburb in coastal area in the Jakarta metropolitan, consists of both agriculture and business areas. A large river crosses the middle of the town; and some areas are commonly flooded during the rainy season. Dengue and typhoid fever are endemic in this area; whereas sporadic outbreaks of influenza H5N1 have been reported since 2005 [5]. This boy had fever for three days (38.0 °C, on admission to the hospital), headache, fatigue, and muscle pain. The patient was alert and anicteric, with blood pressure of 110/70 mmHg, pulse 100 min, and respiratory rate (RR) of 22 min. Complete blood count showed anemia (7.5 mmol/L), thrombocytopenia (68,000 µL), neutrophilia (84 %), elevated absolute neutrophil count (ANC) (6048 µL), and normal leukocyte count (7200 µL). Based on these findings, a diagnosis of dengue hemorrhagic fever grade I was made; and the patient received supportive treatment with intravenous fluid, acetaminophen, and omeprazole.

On the third day of hospitalization, due to persistent fever, thrombocytopenia (20,000 µL), leukocytosis (15,000 µL), decreasing hemoglobin (5.4 mmol/L), and jaundice (total bilirubin/TB 82 µmol/L); the clinical team conducted additional tests which were blood smear and Coomb’s tests for Evans syndrome, dengue NS1 and IgM antibodies, HAV and HCV antibodies, HBsAg, and serum transaminases. All tests, except increased of AST (371 U/L) and ALT (120 U/L), were negative. On the fifth day of hospitalization, the patient appeared septic with oliguria, continued icterus, and RR of 26/min. The clinicians requested rapid IgM test (SD^®^) for leptospira which came back positive. Biochemistry showed increased of TB (366 µmol/L), AST (660 U/L), ALT (179 U/L), BUN (11.1 mmol/L), and creatinine (442 µmol/L).

Leukocytosis and thrombocytopenia were persistent; and metamyelocytes were detected in blood smear. Blood cultures were negative. Interview with the family member revealed that the patient played in flood water near his house a week before illness. Treatment with ceftriaxone 70 mg/kg twice a day was commenced and continued for 14 days.

On the eighth day of hospitalization, the patient’s renal function declined (BUN 20.35 mmol/L, creatinine 503.8 µmol/L); and he became anuric with decreased consciousness (Glasgow Coma Scale/GCS = 7). While waiting for the availability of the intensive care unit (ICU) room for intubation, as required by the hospital standard procedure, the GCS improved in the following day. The patient was hemodialysed on the 10th and 12th day of hospitalization.

After hemodialysis, laboratory parameters began to improve; and on the 21st day of hospitalization, the patient was clinically improved and renal function became normal. The patient was discharged although he was still anemic (Hb 5.5 mmol/L), had TB of 82.4 µmol/L and c-reactive protein (CRP) of 1.2 mg/dL. His hemoglobin and bilirubin returned to normal three weeks after discharge.

With the parents’ consent, serial plasma and serum were stored and later tested to confirm leptospira infection. Leptospira IgM rapid test (SD^®^) was positive in the samples on the first, fifth, and 20th day of hospitalization, and one week after discharge. The micro-agglutination test (MAT) assay was conducted in the leptospira reference center at Kariadi Hospital, Semarang on serum samples from the first, 10th, and 20th day of hospitalization. The titer for *Leptospira serogroup bataviae* increased from <1:20 (on the first day and 10th day) to 1:640 (on the 20th day).

**Fig. 1:**
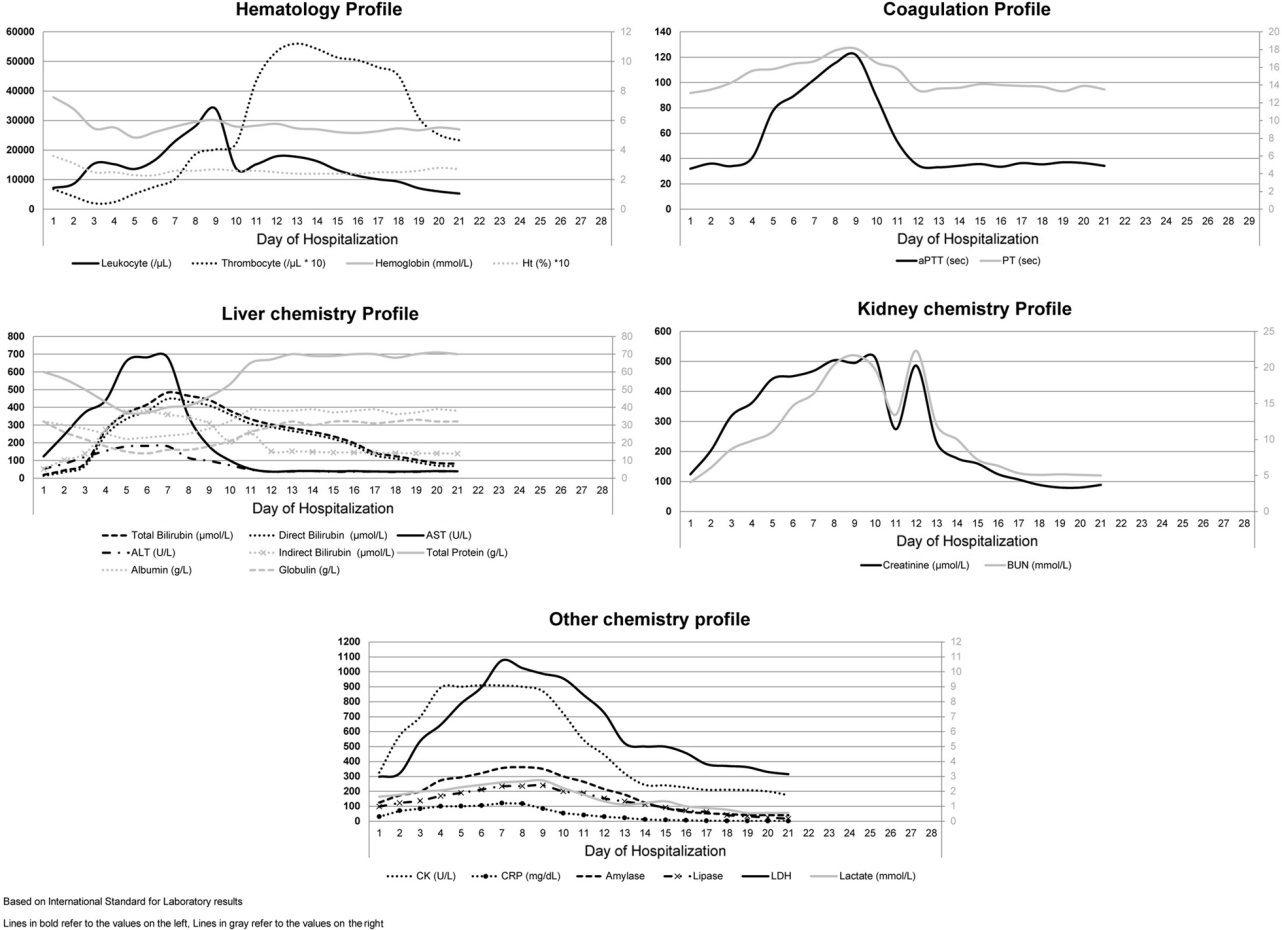
Daily hematology, coagulation and biochemistry profiles

Retrospective tests on plasma samples during admission were negative for dengue NS1, dengue IgM, and *Salmonella typhi* IgM. However, several biochemistry parameters were slightly elevated, which were direct bilirubin (13.7 µmol/L), AST (123 U/L), ALT (53 U/L), BUN (4.0 mmol/L), creatinine (123.8 µmol/L), creatinin-kinase (CK) (326 U/L), CRP (30.5 mg/dL), amylase (125 U/L), and lipase (98 U/L). Serial hematology and biochemistry profiles are described in Fig. 1.

## Conclusions

Our report highlights the importance of considering leptospirosis as a differential diagnosis in children with acute febrile illness; even though the signs and symptoms for more common diagnoses, such as dengue or typhoid fever, were pathognomonic. This case also highlights the need for considering disease specific tests as early as possible to confirm etiology. In case these tests were performed on the admission, a normal leukocyte count with neutrophilia; and negative dengue NS1, dengue IgM, and *Salmonella typhi* IgM should raise suspicion of other infections. This should lead to leptospirosis assays and a full hepatic panel examination.

Unlike in our case report, leptospirosis in children is often milder than in adults [2, 3]. Our literature search only finds a few case reports of children with Weil’s disease or severe leptospirosis [6–9], and none of them was from Indonesia. The involvement of multisystem organs was only reported in the two cases from Samoa and Srilanka [6, 9]; whereas in two other cases, no liver involvement was detected [7, 8].

In our case, an early 2–3 times increase in transaminase, amylase, lipase, and CK on admission indicated hepatic and pancreatic involvement; and rhabdomyolysis. Neutrophilia, ANC, and thrombocytopenia on admission, followed by hemolytic anemia and the appearance of immature erythrocytes and increasing BUN and creatinine level on the second day, indicated hematological abnormalities and acute renal injury. Pulmonary involvement was evident by dyspnea and pleural effusion; and coagulation disorder was evident by increasing prothrombin time (PT) and an activated partial thromboplastin time (PTT) on the fifth day. Neurological involvement occurred on the eighth day of hospitalization when the patient was unconscious (GCS = 7). Platelet was the first that returned to normal (on the eighth day), followed by PT, aPTT, and transaminase (on the 10–12th day).

The earlier recoveries of transaminase and persistent hyperbilirubinemia, which may last for more than five weeks [10], suggest that disruption of hepatocyte intercellular junctions is more severe than hepatocellular damage. The persistence of anemia might be associated with hemolytic uremic syndrome which occurs in rare cases of severe leptospirosis, probably as a result of the action of leptospiral toxins [11]; and/or the disturbance of erythropoietin production due to renal peritubular function impairment [1]. As this patient has no underlying diseases or comorbidities, we can only speculate that (1) the clinical severity was associated with the Batavia strain that was found in this patient, which might be as virulent as other strains that were previously reported (e.g.: strain icterohemorrhagica) [12]; and/or (2) the route of exposure (skin) and infectious dose were the risk factors for severity [13, 14]; (3) severe leptospirosis is rare and therefore the asymptomatic infection and clinically mild cases are rarely confirmed or often misdiagnosed with dengue or salmonella infections [14]; (4) the severity was associated with the delay of the prompt treatment as it is well-documented that appropriate and early antibiotic treatment in leptospirosis cases leads to better disease outcomes [1].

Our report also highlights the importance of having accurate and early diagnostic tests for dengue infection to convince clinicians that when the results are negative, dengue infection can be excluded and other etiologies should be considered. The current dengue NS1 test is very specific for the first few days of fever; however, the sensitivity is low especially in endemic countries [15], whereas the IgM may only be accurate on the fifth day of illness [16].

Despite the detection of leptospira by using Leptospira IgM rapid test (SD^®^) in our case, this rapid test has not been validated. In addition, the confirmation test (MAT) can only be conducted at a reference laboratory, which are only available at two hospitals in Indonesia. Given the lack of reliable rapid tests and often unclear contact and exposure history, the findings of leukocytosis with a high ANC, elevated CK, and raised of hepatic and renal function parameters may suggest leptospirosis. Early diagnosis of leptospirosis would enable clinicians to start antibiotic treatment earlier, which is critical for reducing morbidity and mortality.

## Additional file

10.1186/s13104-016-2210-4 Dataset for the case report.
